# Genetic analysis of late-maturity α-amylase in twelve wheat populations

**DOI:** 10.1007/s00425-023-04319-5

**Published:** 2024-01-24

**Authors:** William Fairlie, Adam Norman, James Edwards, Diane E. Mather, Haydn Kuchel

**Affiliations:** 1 School of Agriculture, Food and Wine, The University of Adelaide, PMB1, Glen Osmond, SA 5064 Australia; 2 Australian Grain Technologies, PO Box 341, Roseworthy, SA 5371 Australia

**Keywords:** Aleurone, Genetic loci, Gibberellic acid signalling, Late maturity α-amylase (LMA), Molecular markers, QTL halotypes, *Rht-D1b*, *Rht13*, *Rht18*

## Abstract

**Main conclusion:**

Genetic loci, particularly those with an effect in the independent panel, could be utilised to further reduce LMA expression when used with favourable combinations of genes known to affect LMA.

**Abstract:**

Late maturity α-amylase (LMA) is a grain quality defect involving elevated α-amylase within the aleurone of wheat (*Triticum aestivum* L.) grains. The genes known to affect expression are the reduced height genes *Rht-B1* (chromosome 4B) and *Rht-D1* (chromosome 4D), and an *ent*-copalyl diphosphate synthase gene (*LMA-1*) on chromosome 7B. Other minor effect loci have been reported, but these are poorly characterised and further genetic understanding is needed. In this study, twelve F_4_-derived populations were created through single seed descent, genotyped and evaluated for LMA. *LMA-1* haplotype C and the *Rht-D1b* allele substantially reduced LMA expression. The alternative dwarfing genes *Rht13* and *Rht18* had no significant effect on LMA expression. Additional quantitative trait loci (QTL) were mapped at 16 positions in the wheat genome. Effects on LMA expression were detected for four of these QTL in a large independent panel of Australian wheat lines. The QTL detected in mapping populations and confirmed in the large independent panel provide further opportunity for selection against LMA, especially if combined with *Rht-D1b* and/or favourable haplotypes of *LMA-1*.

## Introduction

Late maturity α-amylase (LMA) is a grain quality defect in wheat (*Triticum* spp.) (Mares and Mrva 2008; Mrva et al. 2009). This defect results in the synthesis of a high pI α-amylase during the middle to late stages of grain development (Mrva and Mares 2001a). The high-pI α-amylase is not degraded and remains within the grain through to maturity. Grain parcels with high α-amylase activity are associated with a general decline in the quality of non-leavened products (Edwards et al. 1989; Kiszonas et al. 2018; Olaerts et al. 2018; Fairlie et al. 2023). These grain parcels are frequently downgraded at grain elevators, reducing revenue for wheat producers (USDA 2017). Assessment of LMA expression is too expensive for use in early generation selection. This creates a bottleneck at the end of the breeding pipeline, reducing the rate of genetic gain for other important traits. To enable breeders to select against LMA expression earlier in the breeding program, genes associated with LMA expression should be identified and markers developed for selection.

There are few known genetic mechanisms that reduce LMA expression. Expression of LMA is known to be a result of the α-amylase produced from the *TaAMY1* and *TaAMY4* genes (Mieog et al. 2017). The *TaAMY1* gene is also expressed during grain germination and is influenced by the gibberellic acid (GA) signalling pathway (Mrva and Mares 1999; Mieog et al. 2017). Another gene associated with LMA expression was identified by Derkx et al. (2021) and designated *LMA-1*. The *LMA-1* gene, which is on the long arm of chromosome 7B, encodes an enzyme that is responsible for converting geranylgeranyl diphosphate to *ent*-copalyl diphosphate at the start of the GA signalling pathway. Several haplotypes of the *LMA-1* gene were associated with low LMA expression. One of these, haplotype C, is in linkage disequilibrium with a boron tolerance allele at the *Bo1* boron tolerance locus (Derkx et al. 2021). LMA expression is also reduced by the *Rht-B1b*, *Rht-D1b* and *Rht-B1c* alleles, which have mutations that prevent the binding of GA complexes to the DELLA protein (Mrva and Mares 1996; Middleton et al. 2012). Unlike many other dwarfing mutants, DELLA mutants are not GA responsive (Ellis et al. 2004).

The non-DELLA dwarfing genes *Rht13* and *Rht18* act by diverting GA out of the GA signalling pathway (Rebetzke et al. 2011; Ford et al. 2018). The non-DELLA dwarfing genes are GA-responsive; application of exogenous GA increases plant height (Ellis et al. 2004). *Rht13* and *Rht18* are of interest to wheat breeders as alternatives to *Rht-B1b* and *Rht-D1b* for the development of semi-dwarf wheat varieties with improved early vigour (Ellis et al. 2004). However, it is not known what effect the GA-sensitive dwarfing genes have on LMA expression.

The aim of the research reported here was to investigate the effects of *LMA-1* haplotype C (*LMA-1-C*), *Rht-D1b*, *Rht13* and *Rht18* on LMA expression, and to discover other genetic loci that could be used to select against LMA expression. This was performed in three stages, firstly, the evaluation of known genes in seven populations, secondly, quantitative trait loci (QTL) analysis in five populations and thirdly, further investigation of QTL in a panel of Australian wheat lines.

## Materials and methods

### Plant material

Twelve F_4_-derived populations, with the number of lines tested in each population in brackets (Hal/875 (80), Hal/Esp (129), Hal/Kat (79), Hal/Sce (90), Kat/Sce (112), M13/Sce (139), M18/875 (40), M18/Esp (90), M18/Hal (40), M18/Kat (40), M18/Ken (40) and M18/Sce (132); Tables 1 and 2), were produced through crossing and four generations of single seed descent. The purpose of the M13/Sce, M18/875, M18/Esp, M18/Hal, M18/Kat, M18/Ken and M18/Sce populations (Table 1) was for the evaluation of known genes, while Hal/875, Hal/Esp, Hal/Kat, Hal/Sce and Kat/Sce (Table 2) were used to map new QTL. The use of M18 (Magenta-Rht18) across several populations was to assess its effect in different genetic backgrounds, while Hal (Halberd) was used as a common parent due to its tall genotype and low LMA expression. Lines were genotyped using markers linked with *Bo1* and *LMA-1-C* (Pallota et al. 2014), *Rht-D1b* (Ellis et al. 2002), *Rht13* (Borrill et al. 2022) and *Rht18* (Ford et al. 2018) in the populations known to be segregating for the particular gene. The genotypic data were used to select homozygous genotypes, tall genotypes in four populations (Hal/875, Hal/Esp, Hal/Kat and Hal/Sce, Table 2), lines without *LMA-1-C* in one population (Kat/Sce, Table 2) and to help correct for segregation distortion at the above loci in seven populations (M13/Sce, M18/875, M18/Esp, M18/Hal, M18/Kat, M18/Ken and M18/Sce, Table 1). The purpose of selection for tall genotypes or against *LMA-1-C* was to avoid the large effect of *Rht-D1b* or *LMA-1-C* from masking loci of small effect in the QTL analysis. Selected lines were genotyped using the custom Axiom™ Affymetrix array containing 18,101 single nucleotide polymorphism markers as described by Norman et al. (2017). Further selection against highly related lines was applied prior to the inclusion of lines in the experiment.

**Table 1:** Frequencies of genotype combinations within each of the seven wheat populations derived from crosses involving *LMA-1* haplotype C (*LMA-1-C*), *Rht-D1b*, *Rht13* and *Rht18*

Population	Pedigree	Initial population size	No. of lines tested	No. of lines homozygous for alleles reducing LMA or plant height										
				*LMA-1-C*	*Rht-D1b*	*Rht13*	*Rht18*	*LMA-1-C* and *Rht-D1b*	*LMA-1-C* and *Rht13*	*LMA-1-C* and *Rht18*	*Rht-D1b* and *Rht13*	*Rht-D1b* and *Rht18*	*LMA-1-C* and *Rht-D1b* and *Rht13*	*LMA-1-C* and *Rht-D1b* and* Rht18*
M13/Sce	Magenta Rht13/Scepter	556	139	67	20	77	0	9	8	0	10	0	1	0
M18/875	Magenta Rht18/RAC875	184	40	0	12	0	18	0	0	0	0	6	0	0
M18/Esp	Magenta Rht18/Espada	370	90	52	27	0	36	19	0	19	0	11	0	6
M18/Hal	Magenta Rht18/Halberd	184	40	23	0	0	17	0	0	12	0	0	0	0
M18/Kat	Magenta Rht18/AGT Katana	184	40	0	13	0	16	0	0	0	0	4	0	0
M18/Ken	Magenta Rht18/Kennedy	184	40	0	13	0	10	0	0	0	0	2	0	0
M18/Sce	Magenta Rht18/Scepter	556	132	77	13	0	37	7	0	20	0	4	0	3

**Table 2:** Frequencies of lines homozygous for *LMA-1* haplotype C (*LMA-1-C*) or the *Rht-D1b* allele in five wheat populations after selection against *LMA-1-C* or *Rht-D1b*

Population	Pedigree	Allele selected against	Initial population size	No. of lines tested	No. of lines homozygous for alleles previously shown to reduce LMA	
					*LMA-1-C*	*Rht-D1b*
Hal/875	Halberd/RAC875	*Rht-D1b*	556	80	44	0
Hal/Esp	Halberd/Espada	*Rht-D1b*	556	129	129	0
Hal/Kat	Halberd/AGT Katana	*Rht-D1b*	556	79	54	0
Hal/Sce	Halberd/Scepter	*Rht-D1b*	370	90	90	0
Kat/Sce	AGT Katana/Scepter	*LMA-1-C*	556	112	0	112

Plants were grown in accordance with findings reported by Derkx and Mares (2020), which indicated that a cool shock is not essential to induce LMA expression. The plants were grown in a mild, stress-free environment, under natural light with a peak photosynthetic flux measured at approximately 1200 µmol m^−2^ s^−1^. The cooling target for the evaporative cooling was set at 25 °C. The primary culm was harvested, oven-dried at 40 °C for three days and the grain threshed out using a Wintersteiger LD180 laboratory thresher (Wintersteiger AG, Ried, Austria). Grain was then stored in cool (11 °C) conditions until required in the high-pI α-amylase enzyme-linked immunosorbent assay (ELISA).

### High-pI α-amylase enzyme-linked immunosorbent assay (ELISA)

Grain from each sample was milled using a Perten 3310 laboratory mill fitted with a type-2 fine disc (PerkinElmer Co., Hägersten, Sweden). Grist of each sample (200 mg ± 4 mg) was aliquoted into 96-well 2 mL titre plates for extraction, extracted using 1 mL of 0.5% NaCl and 0.02% CaCl_2_ extraction buffer per well and incubated for 16 h at 37 °C at 100 motions min^−1^. The extraction plate was then centrifuged at 2000 g for 10 min. An ELISA specific to high-pI α-amylase (SARDI, Urrbrae, SA, Australia) was then performed (Verity et al. 1999).

### Genetic maps

Linkage maps were constructed for five of the populations (Hal/875, Hal/Esp, Hal/Kat, Hal/Sce and Kat/Sce, Table 2) based on genetic data for the known loci (*LMA-1-C*, *Rht-D1b*, *Rht13* and *Rht18*) and data from the 18,101-feature single nucleotide polymorphism array. All mapping work was carried out using the R/ASMap package (Taylor and Butler 2017), which draws on functionality from R/qtl (Broman et al. 2003). Lines with missing genotype calls for more than 2000 markers were removed. For pairs or groups of lines with genetic similarity greater than 98%, marker data were merged into a single consensus genotype for linkage mapping. Monomorphic markers, markers with greater than 20% missing calls and markers that had a minor allele frequency less than 25% were removed. The remaining high-quality markers were then distributed to linkage groups associated with each chromosome using the *mstmap* algorithm (Wu et al. 2008). Linkage groups with fewer than eight markers were then removed. Lines that had high rates of crossovers, double crossovers or missing marker data were also removed. The markers were then remapped within their assigned linkage groups and genetic distances estimated using the R/ASMap *quickEst* function. Linkage groups were then compared by chromosome, with alleles switched for linkage groups that were out of phase. Linkage groups were then merged by chromosome and the markers remapped at the chromosome level and genetic distances re-estimated.

### Experiment design and analysis

#### Experiment design

The glasshouse experiment was designed using the R/odw package (Butler and Cullis 2018) in the R statistical computing environment (R Core Team 2023). The 1024 entries included lines from each of the twelve populations, the parents of the populations and two additional control lines (RAC655 and Seri 82). A randomised complete block design consisting of three replicate blocks of 32 rows and 32 columns was used (Bailey 2008). The design was randomised according to the treatment, *Line*. The observational units in the glasshouse phase of the experiment were 3072 cells spread across multiple seed-raising trays, and each observational unit (OU1) could be completely indexed by *Column* and *Row*.

The ELISA phase of the experiment was designed using R/odw with a replication level of 33%. The multiphase design accounted for the design factors of the distinct glasshouse and laboratory ELISA phases of the experiment (Smith et al. 2006). The experiment was designed such that OU1’s with multiple observational units in the ELISA phase were resolvable across blocks of 21 ELISA plates (*Wblock*). The design was randomised according to the treatment and treatment factor, *OU1* and *Block*. The observational units in the ELISA phase were 4032 wells spread across 42 ELISA plates, and could be completely indexed by *Plate*, *Wcolumn* and *Wrow*.

#### Data preparation and base analysis

Data checking and fitting of linear mixed models were performed in R/ASReml (Gilmour et al. 1997; Butler et al. 2018; R Core Team 2023). Given the highly skewed distribution of the optical density (OD) data, a generalised logistic transformation (logitOD) accounting for the minimum and maximum OD values was considered appropriate.

$$logitOD={\text{ln}}\left(\frac{\left(ELISA OD-0.04\right)}{\left(3.50-ELISA OD\right)}\right)$$

Studentised conditional residuals were then used to help identify any outliers, with a single data point removed. The design factors for the observational units of the second phase of the experiment were drawn from the first (*Block*, *Column*, *Row* and *OU1*) and second phase (*Wblock*, *Plate*, *Wcolumn* and *Wrow*) and included as random effects. Variance for *Wcolumn* and *Wrow* was investigated at the *Plate* level to allow for different levels of variance in each plate, given that each plate was processed individually.

#### Evaluation of the effects of known genes

The effect of *LMA-1* (haplotypes C and F), *Rht-D1* (*Rht-D1a* and *Rht-D1b*), *Rht13* and *Rht18* on LMA expression was assessed in each population in which the gene was segregating (Table 1). The genes were evaluated in a joint analysis across seven populations (M13/Sce, M18/875, M18/Esp, M18/Hal, M18/Kat, M18/Ken and M18/Sce) using R/ASReml (Henderson 1953; Butler et al. 2018). The genes and gene interactions included as fixed effects were *LMA-1*, *Rht-D1*, *Rht13*, *Rht18*, *LMA-1:Rht-D1*, *LMA-1:Rht13*, *LMA-1:Rht18*, *Rht-D1:Rht13*, *Rht-D1:Rht18* and *LMA-1:Rht-D1:Rht18*. Genetic data for markers within 20 cM exclusion windows for the *LMA-1*, *Rht-D1*, *Rht13* and *Rht18* genes was removed prior to derivation of the k-matrix (Taylor and Verbyla 2011). The random model contained the k-matrix and *Line* to partition the additive and residual genetic effects, respectively (Forni et al. 2011). Design factors relevant to the first (*Block*, *Column*, *Row*, *OU1*) and second phase (*Wblock*, *Plate*, *Wcolumn* and *Wrow*) of the experiment were also included in the random model (Bailey 2008). In the final model only the significant genes *LMA-1* and *Rht-D1* and the significant interaction *LMA-1:Rht-D1* were included, along with a k-matrix derived with 20 cM exclusion windows for *LMA-1* and *Rht-D1*.

#### QTL analysis

The package R/wgaim (Taylor and Verbyla 2011) was used in conjunction with the baseline linear mixed model to detect significant QTL intervals in five populations (Hal/875, Hal/Esp, Hal/Kat, Hal/Sce and Kat/Sce, Table 2). For the baseline linear mixed model using R/ASReml, *Population* was included as a fixed effect. The random model contained *Line* (partitioned by population to allow the QTL search to be performed by population), in addition to the design factors relevant to the first (*Block*, *Column*, *Row* and *OU1*) and second phase (*Wblock*, *Plate*, *Wcolumn* and *Wrow*) of the experiment (Bailey 2008). The QTL search was performed for each population using R/wgaim, where the familywise α-level of significance was set at 0.05, the two flanking markers of the QTL are identified rather than the most likely marker, and an exclusion window of 20 cM applied to the genetic data near the flanking markers of each QTL. The exclusion window was applied after a QTL was identified and applied for the remainder of the analysis. The physical positions of the flanking markers for each QTL were obtained through a BLAST search of the Chinese Spring International Wheat Genome Sequencing Consortium RefSeq v2.1 genome assembly (Zhu et al. 2021) via https://wheat.pw.usda.gov/blast/ and are reported for ease of comparison with previously identified QTL.

### Investigation of QTL in a breeding panel

A panel comprising of 614 Australian wheat varieties and breeding lines that had previously been genotyped with the 18,101-feature single nucleotide polymorphism array and assessed for LMA expression (2019–2021, best linear unbiased predictors for OD from each year) (Butler et al. 2009) using the glasshouse-based cool shock method of Mrva and Mares (2001a) was used for investigation of identified QTL. Haplotypes were assigned based on the data for the two flanking markers of each QTL interval, with the potential for two parental and two recombinant haplotypes to be assigned for each QTL. The two parental haplotypes were used for QTL investigation. An initial k-matrix was developed using markers that were not within 20 cM of any QTL interval (Taylor and Verbyla 2011). The random model contained the treatment *Line*, in conjunction with a k-matrix to partition the additive and residual genetic effects (Forni et al. 2011), and the treatment factor *Year*. The fixed model consisted of all identified QTL. QTL with no significant effect in the panel were then removed from the fixed model, replaced by the interactions of the four remaining (significant) QTL and the analysis re-run with an updated k-matrix based on markers that were not within 20 cM of the remaining QTL.

## Results

### Effects of known genes

Among the lines used for evaluating the effects of known genes, LMA expression ranged from -5.5 to 2.0 logitOD. The broad-sense heritability of the analysis was 0.87 and the narrow-sense heritability 0.69, as calculated from Suppl. Table S1. The *Rht-D1b* allele of the reduced height gene was associated with a reduction in LMA expression of 1.86 logitOD units when compared to *Rht-D1a* (Table 3). *LMA-1-C*, which is carried by Espada, Halberd and Scepter, was associated with a reduction in LMA expression of 1.90 logitOD units, compared to *LMA-1* haplotype F, which is carried by AGT Katana, Kennedy, Magenta Rht13, Magenta Rht18 and RAC875. The effects of *LMA-1* and *Rht-D1* were only partially additive, with the combination of *Rht-D1b* and *LMA-1-C* having a genotypic effect of 1.13 logitOD, contributing to an overall reduction in LMA expression of 2.63 logitOD units when compared to the combination of *Rht-D1a* and *LMA-1* haplotype F. The alternative dwarfing genes *Rht13* and *Rht18* had no significant effect on LMA expression, either alone or in combination with *LMA-1* or *Rht-D1*.

**Table 3:** Genotypic effects for LMA expression in populations (M13/Sce, M18/875, M18/Esp, M18/Hal, M18/Kat, M18/Ken and/or M18/Sce) where the gene or gene combination was segregating

Gene	Effect
*LMA-1*	− 1.90 ***
*Rht-D1*	− 1.86 ***
*Rht13*	n.s
*Rht18*	n.s
*LMA-1*:*Rht-D1*	1.13 ***
*LMA-1*:*Rht13*	n.s
*LMA-1*:*Rht18*	n.s
*Rht-D1*:*Rht13*	n.s
*Rht-D1*:*Rht18*	n.s
*LMA-1*:*Rht-D1*:*Rht13*	n.s
*LMA-1*:*Rht-D1*:*Rht18*	n.s

*LMA-1-C* is being tested against *LMA-1* haplotype F, while *Rht-D1b* is being tested against *Rht-D1a*

^***^Significant at ≤ 0.001 level of error probability, n.s. non-significant at ≤ 0.05 level of error probability, gene interaction; *LMA-1-C*, *LMA-1* haplotype C

### QTL analysis for mapping populations

The QTL interval analysis performed in five mapping populations (Hal/875, Hal/Esp, Hal/Kat, Hal/Sce and Kat/Sce, Table 2) detected 17 QTL across 13 of the 21 wheat chromosomes (Table 4), where the two markers flanking the QTL and their RefSeq v2.1 physical position is presented. These QTL accounted for between 2.9 and 77% of the genetic variance, with absolute effects ranging from 0.22 to 1.07 logitOD units. Of the 17 QTL, only two (*QLMA.agt-7B.1* in Hal/Kat and *QLMA.agt-7B.2* in Hal/875) collocated with each other, near *LMA-1* on chromosome 7B. Each of these 7B QTL explained more than 70% of the genetic variance, with the low LMA haplotype inherited from Halberd, which carries *LMA-1-C*. The 7B locus was not identified in other populations being analysed for QTL as they were homozygous for *LMA-1* haplotype F (Table 2)*.* At the QTL on other chromosomes, the low LMA haplotypes were inherited from AGT Katana (*QLMA.agt-1B*, *QLMA.agt-3D*, *QLMA.agt-4B.1* and *QLMA.agt-7A.1*), Espada (*QLMA.agt-2B*), Halberd (*QLMA.agt-2A.1*, *QLMA.agt-3A*, *QLMA.agt-3B.2*, *QLMA.agt-4B.2*, *QLMA.agt-5A*, *QLMA.agt-5B* and *QLMA.agt-7D*), RAC875 (*QLMA.agt-2A.2 QLMA.agt-2D QLMA.agt-3B.1*) and Scepter (*QLMA.agt-7A.2*).

**Table 4:** Position and effect of QTL for LMA expression from whole-genome average interval mapping for the five mapping populations

Chromosome	Population	Flanking markers and RefSeq v2.1 physical positions (Mb)				Effect (logitOD units)	Genetic variance accounted for (%)	LOD	QTL name	*P-*value	Low LMA haplotype
1B	Kat/Sce	AXAFFSNP-12098	335.26	AXAFFSNP-02777	355.14	0.35	13.1	2.607	*QLMA.agt-1B*	0.0005	AGT Katana
2A	Hal/Sce	AXAFFSNP-01894	63.37	AXAFFSNP-06941	64.34	0.31	13.3	2.268	*QLMA.agt-2A.1*	0.0012	Halberd
2A	Hal/875	AXAFFSNP-06874	771.34	AXAFFSNP-05128	780.90	0.43	11.4	7.278	*QLMA.agt-2A.2*	< 0.0001	RAC875
2B	Hal/Esp	AXAFFSNP-01030	775.69	AXAFFSNP-07085	769.88	0.30	35.4	5.300	*QLMA.agt-2B*	< 0.0001	Espada
2D	Hal/875	AXAFFSNP-07562	537.82	AXAFFSNP-13113	532.51	0.22	2.9	1.843	*QLMA.agt-2D*	0.0036	RAC875
3A	Hal/875	AXAFFSNP-00462	552.79	AXAFFSNP-07736	568.59	0.26	4.1	2.587	*QLMA.agt-3A*	0.0006	Halberd
3B	Hal/875	AXAFFSNP-08487	86.57	AXAFFSNP-03929	92.32	0.24	3.4	1.972	*QLMA.agt-3B.1*	0.0026	RAC875
3B	Hal/875	AXAFFSNP-00569	780.92	AXAFFSNP-13586	781.29	0.23	3.4	2.013	*QLMA.agt-3B.2*	0.0023	Halberd
3D	Kat/Sce	AXAFFSNP-13862	444.17	AXAFFSNP-04972	452.50	0.37	14.7	2.638	*QLMA.agt-3D*	0.0005	AGT Katana
4B	Kat/Sce	AXAFFSNP-14371	22.93	AXAFFSNP-09087	24.29	0.40	17.8	3.078	*QLMA.agt-4B.1*	0.0002	AGT Katana
4B	Hal/Sce	AXAFFSNP-09073	641.95	AXAFFSNP-01491	646.75	0.30	12.3	2.303	*QLMA.agt-4B.2*	0.0011	Halberd
5A	Hal/Sce	AXAFFSNP-04886	575.60	AXAFFSNP-09286	586.96	0.51	35.5	5.976	*QLMA.agt-5A*	< 0.0001	Halberd
5B	Hal/Esp	AXAFFSNP-09665	660.24	AXAFFSNP-03751	673.87	0.31	37.5	4.932	*QLMA.agt-5B*	< 0.0001	Halberd
7A	Hal/Kat	AXAFFSNP-11274	18.22	AXAFFSNP-11213	18.35	0.33	9.2	3.003	*QLMA.agt-7A.1*	0.0002	AGT Katana
7A	Hal/Sce	AXAFFSNP-11236	82.16	AXAFFSNP-02196	87.94	0.41	23.1	4.373	*QLMA.agt-7A.2*	< 0.0001	Scepter
7B	Hal/Kat	AXAFFSNP-16522	725.98	AXAFFSNP-11482	727.44	0.94	77.0	21.601	*QLMA.agt-7B.1*	< 0.0001	Halberd
7B	Hal/875	AXAFFSNP-16522	725.98	AXAFFSNP-11596	735.83	1.07	70.4	43.917	*QLMA.agt-7B.2*	< 0.0001	Halberd
7D	Hal/875	AXAFFSNP-04066	48.77	AXAFFSNP-11816	47.36	0.23	3.3	2.114	*QLMA.agt-7D*	0.0018	Halberd

*LOD* likelihood of differences

### Investigation of QTL in a breeding panel

Data for LMA expression from the Australian wheat industry LMA screen was used to further investigate effects of the 17 QTL positions listed in Table 4. Only four of these QTL had significant effects in the panel of Australian wheat lines, as shown in Table 5. The QTL on chromosome 7B had the largest effect, with the Halberd haplotype contributing a large reduction in LMA expression (0.36 optical density, OD). *QLMA.agt-7A.1* had a moderate effect, with the Halberd haplotype resulting in a slight decrease (0.25 OD) in LMA expression. *QLMA.agt-3A* also had a moderate effect, with the Halberd haplotype decreasing LMA expression by 0.20 OD. *QLMA.agt-2B* had a small effect, with the Halberd haplotype decreasing LMA expression by 0.11 OD relative to the Espada haplotype. The combination of the *QLMA.agt-2B* and *QLMA.agt-7A.1* QTL had a small effect, with the interaction of the AGT Katana and Espada low-LMA donor haplotypes for each respective QTL reducing LMA expression by 0.24 OD relative to the Halberd haplotype. However, an overall increase in LMA expression of 0.12 OD was observed for the interaction after accounting for the individual effect of each QTL haplotype.

**Table 5:** Frequency and effect (optical density, OD) of the two parental QTL haplotypes as a source of low- and high-LMA expression in a large Australian wheat panel previously screened for LMA (2019–2021 industry LMA screen, *n* = 684)

QTL	Low-LMA donor(s)	High-LMA donor	No. of homozygous lines		Effect (OD)
			Low-LMA haplotype	High-LMA haplotype	
*QLMA.agt-2B*	Espada	Halberd	238	70	0.11*
*QLMA.agt-3A*	Halberd	RAC875	195	60	– 0.20*
*QLMA.agt-7A.1*	AGT Katana	Halberd	118	71	0.25**
*QLMA.agt-7B.1*	Halberd	AGT Katana	246	383	− 0.36***
*QLMA.agt-2B* and* QLMA.agt-7A.1*	Espada and AGT Katana	Halberd	17	58	− 0.24*

All other loci from Table 4 were examined and found to be non-significant

^*^, **, *** Significant at ≤ 0.05, ≤ 0.01, and ≤ 0.001 levels of error probability, respectively

## Discussion

The reduction in LMA expression associated with *LMA-1-C* and *Rht-D1b* (Table 3) is consistent with previous observations (Mrva and Mares 1996, 2002; Derkx et al. 2021). The *ent*-copalyl diphosphate synthase gene at the *LMA-1* locus may affect LMA via the GA biosynthesis pathway (Derkx et al. 2021). Meanwhile, the *Rht-D1b* allele affects the functionality of DELLA in the GA-signalling pathway (Peng et al. 1999; Hartweck and Olszewski 2006), ultimately reducing both plant height and LMA expression (Mrva and Mares 1996; Ellis et al. 2004). In contrast, the alternative dwarfing genes *Rht13* and *Rht18*, which divert GA out of the GA biosynthesis pathway (Rebetzke et al. 2011; Ford et al. 2018), did not reduce LMA expression despite producing a similar reduction in height to that of *Rht-D1b* (Ellis et al. 2004). The alternative dwarfing genes *Rht13* and *Rht18* have been proposed as potential replacements for *Rht-B1b* and *Rht-D1b*, which are widely used to reduce plant height, but also reduce early vigour (Ellis et al. 2004). Given that LMA expression can be reduced by *Rht-B1b* and *Rht-D1b* (Mrva and Mares 1996), wheat breeders will have to balance increasing early vigour against the risk of increasing LMA expression in deciding whether to use the *Rht13* and *Rht18* dwarfing genes as an alternative source of height reduction over *Rht-B1b* or *Rht-D1b*.

Several QTL were found to affect LMA expression and are of potential interest for genetic selection against LMA. However, these QTL had much smaller effect than the QTL associated with the *LMA-1* gene located on chromosome 7B (Table 4). The lack of QTL in common between populations could be due to monomorphic regions, relatively small sample size or error variance. Based on its estimated physical position on chromosome 1B (335.3 Mb), *QLMA.agt-1B*, which had a moderate effect on LMA expression, seems to differ from the 1B/1R translocation (at 25.5 Mb) which affects falling number and α-amylase content (Farrell et al. 2013; Mohler et al. 2014). *QLMA.agt-1B* is also different to QTL for α-amylase content reported by Yang et al. (2014) (460.0 Mb) and Mares et al. (2023) (123.7 Mb).

*QLMA.agt-2A.1* and *QLMA.agt-2A.2* both have small effects on LMA expression. The estimated physical position of *QLMA.agt-2A.1* (63.4 Mb) is close to the estimated physical position of one of two LMA QTL (76.1 Mb and 130.5 Mb) reported by Mares et al. (2023). *QLMA.agt-2A.2* (at 771.3 Mb) is not near any previously reported QTL associated with LMA. *QLMA.agt-2B* has a small effect on LMA expression. At an estimated position of 769.9 Mb, it is closer to a QTL reported by Mares et al. (2023) (726.1 Mb) than to one reported by Zhang et al. (2014) (677.4 Mb). *QLMA.agt-2D* has a small effect on LMA expression. A QTL has previously been identified on chromosome 2D for α-amylase content (Tan et al. 2010). However, this QTL (647.1 Mb) is not close to the *QLMA.agt-2D* QTL (532.5 Mb).

*QLMA.agt-3A* has a small effect on LMA expression. QTL have previously been identified on chromosome 3A for α-amylase content (Verbyla and Cullis 2012; Liu et al. 2021; Mares et al. 2023). However, the QTL (7.5 Mb, 24.5 Mb and 654.1 Mb) are not close to *QLMA.agt-3A* (552.8 Mb). *QLMA.agt-3B.1* and *QLMA.agt-3B.2* have moderate effects on LMA expression. Their estimated physical positions are 86.6 Mb and 780.9 Mb, respectively. While *QLMA.agt-3B.1* could correspond with a QTL that Mares et al. (2023) detected at 115.6 Mb, neither of these loci are near QTL reported by Tan et al. (2010) (12.5 Mb), Mares et al. (2023) (506.9 Mb) or Mrva and Mares (2001b) (516.2 Mb).

*QLMA.agt-4B.1* and *QLMA.agt-4B.2* have moderate effects on LMA expression. While *QLMA.agt-4B.1* (22.9 Mb) could correspond with *Rht-B1*, which is located at 33.6 Mb, the fact that Scepter carries the *Rht-B1a* allele indicates that the loci are not coincident.

*QLMA.agt-5A* and *QLMA.agt-5B* both have a moderate effect on LMA expression. Based on the physical position of *QLMA.agt-5A* (575.6 Mb), it is not near any previously reported QTL for FN (Zhang et al. 2014; Börner et al. 2018; Martinez et al. 2018) or the *TaAMY3* and *TaAMY4* genes (645.7 Mb and 455.3 Mb, respectively) (Mieog et al. 2017). Meanwhile, *QLMA.agt-5B* (660.2 Mb) could correspond with a QTL that Tan et al. (2010) and Verbyla and Cullis (2012) detected at 623.8 Mb, or the *TaAMY3* gene (Mieog et al. 2017) located at 648.6 Mb. However, none of these loci are near the *TaAMY4* gene located at 420.7 Mb.

*QLMA.agt-7A.1* and *QLMA.agt-7A.2* both have a moderate effect on LMA expression. Their estimated physical positions are 18.2 Mb and 82.2 Mb, respectively. While *QLMA.agt-7A.1* could correspond with a QTL identified by Martinez et al. (2018) (0.5 Mb), neither of these loci are close to a QTL reported by Liu et al. (2021) (602.7 Mb). *QLMA.agt-7B.1* and *QLMA.agt-7B.2* are collocating and have a large effect on LMA expression. The locus has previously been designated *LMA-1* (Derkx et al. 2021) and has had a large effect in several studies investigating α-amylase content or FN (Mrva and Mares 2001b; Mrva et al. 2009; Emebiri et al. 2010; Mohler et al. 2014; Zhang et al. 2014; Börner et al. 2018; Martinez et al. 2018; Liu et al. 2021). *QLMA.agt-7D* has a small effect on LMA expression. QTL have been identified on chromosome 7D for α-amylase content (Liu et al. 2021; Mares et al. 2023). While *QLMA.agt-7D* (47.4 Mb) could correspond with the QTL identified by Liu et al. (2021) (56.6 Mb), neither of these loci are close to the QTL identified by Mares et al. (2023) located at 14.2 Mb.

The *LMA-1* region of chromosome 7B, which has previously been reported to have a large effect on LMA expression and FN (Mrva and Mares 2001b; Mrva et al. 2009; Emebiri et al. 2010; Mohler et al. 2014; Zhang et al. 2014; Börner et al. 2018; Martinez et al. 2018; Liu et al. 2021), had consistently large effects on LMA expression in four of six populations that segregate for *LMA-1-C* (Tables 3 and 4) and had a large effect in the panel of Australian wheat lines (Table 5). *QLMA.agt-2B*, *QLMA.agt-3A* and *QLMA.agt-7A.1* all had a moderate effect on LMA expression in mapping populations (Table 4) and were found to have a similar effect on LMA expression in an Australian wheat panel (Table 5). The combination of *QLMA.agt-2B* and *QLMA.agt-7A.1* was found to be effective and could be pyramided to assist in reducing LMA. The four loci (*QLMA.agt-2B*, *QLMA.agt-3A*, *QLMA.agt-7A.1* and *QLMA.agt-7B.1*) identified in Table 5 warrant further investigation to assess their potential for early selection against LMA in wheat breeding programs.

The greatest opportunities for wheat breeders to select against LMA are in marker-assisted selection, given the costly and destructive nature of LMA phenotyping which limits screening to the late stages of the breeding pipeline. Wheat breeders could also implement genomic selection provided a large enough training dataset is available. The evaluation of *LMA-1-C* and *Rht-D1b* confirms their potential for widespread implementation of marker-assisted selection against LMA expression. Unfortunately, the *Rht13* and *Rht18* dwarfing genes do not reduce LMA expression. This is consistent with their differential effect on the GA-signalling pathway compared to *Rht-B1b* and *Rht-D1b*. Therefore, wheat breeders will have to consider the implications on LMA expression when considering using these alternative dwarfing genes in place of *Rht-B1b* or *Rht-D1b* for height reduction. The *QLMA.agt-2B*, *QLMA.agt-3A* and *QLMA.agt-7A.1* QTL detected here in mapping populations and confirmed in a large panel of Australian wheat lines provide a further opportunity for selection against LMA, especially if pyramided and/or combined with *Rht-D1b* and/or favourable haplotypes of *LMA-1*.

## Data Availability

The datasets generated during and/or analysed during the current study are available in the Figshare repository, [10.25909/23160164].

## Abbreviations

GA: Gibberellic acid
LMA: Late maturity α-amylase
LogitOD: Log-transformed optical density
OD: Optical density
QTL: Quantitative trait loci
